# Experimental Study on the Influence of Inter-Layer Ironing Parameters on the Surface Quality of MEX-Fabricated PLA-LW and PLA Parts

**DOI:** 10.3390/ma19143061

**Published:** 2026-07-16

**Authors:** Ioan Tamașag, Costică Bejinariu, Traian-Lucian Severin, Ștefan-Constantin Lupescu, Marius-Constantin Beniuga, Delia-Aurora Cerlincă, Irina Beșliu-Băncescu, Adrian-Constantin Sachelarie, Gabriel-Dragos Vasilescu, Nicanor Cimpoesu

**Affiliations:** 1 Faculty of Mechanical Engineering, Automotive and Robotics, Stefan cel Mare University, 720229 Suceava, Romania; ioan.tamasag@usm.ro (I.T.); severin.traian@usm.ro (T.-L.S.); stefan.lupescu@usv.ro (Ș.-C.L.); marius.beniuga@usv.ro (M.-C.B.); delia@usm.ro (D.-A.C.); irina.besliu@usm.ro (I.B.-B.); 2 Faculty of Materials Science and Engineering, “Gheorghe Asachi” Technical University of Iasi, 41 Dimitrie Mangeron Blvd., 700050 Iasi, Romania; nicanor.cimpoesu@academic.tuiasi.ro; 3 Academy of Romanian Scientists, Ilfov 3, 050044 Bucharest, Romania; 4 Faculty of Mechanics, “Gheorghe Asachi” Technical University of Iasi, 41 Dimitrie Mangeron Blvd., 700050 Iasi, Romania; adrian-constantin.sachelarie@academic.tuiasi.ro; 5 National Institute for Research and Development in Mine Safety and Protection to Explosion—INSEMEX, 332047 Petrosani, Romania

**Keywords:** additive manufacturing, surface roughness, material extrusion, porosity, shore D hardness, interlayer ironing, PLA-LW, PLA, ultrasonic testing

## Abstract

The current trend in both academic research and industrial applications is to expand the use of additive manufacturing processes across an increasing number of functional domains. This has led to substantial efforts aimed at improving the overall performance of additively manufactured components, particularly those produced by Material Extrusion (MEX). One of the main limitations of MEX-fabricated parts is the presence of air voids formed between deposited lines and layers, which reduce mechanical strength and structural uniformity. In this context, the present study investigates the influence of inter-layer ironing process parameters, an approach intended to modify near-surface morphology and improve the surface quality and dimensional accuracy of two commonly used materials, namely lightweight polylactic acid (PLA-LW) and standard. The experimental setup involved varying the ironing direction, nozzle diameter, and ironing spacing, while keeping all other manufacturing parameters constant. A complete 4 × 3 × 3 factorial experimental design was employed, considering three process parameters: ironing direction at four levels, nozzle diameter at three levels, and ironing spacing at three levels, resulting in 36 parameter combinations applied to each material separately. Performance evaluation included surface roughness (S_a_), Shore D hardness, waviness (W_a_), microscopic morphology analysis, and qualitative ultrasonic inspection used to observe internal void-related features, whereas the quantitative analysis focused on Sa, Wa, and hardness variations. The results were compared with those obtained for specimens produced using final-layer-only ironing and no ironing. Inter-layer ironing generally improved surface quality compared with non-ironed specimens, reducing Sa by up to 85.22% and increasing Shore D hardness by up to 10.40% for individual PLA-LW specimens manufactured using the 0.4 mm nozzle. The waviness response was strongly material-dependent; increasing the ironing spacing from 0.1 to 0.3 mm reduced Wa by 47.71% for PLA-LW and 13.39% for standard PLA, while the same spacing increase reduced Sa by 25.61% for PLA-LW but increased Sa by 10.87% for standard PLA. While PLA-LW specimens exhibited localized surface defects possibly associated with the compaction or collapse of near-surface voids, standard PLA specimens showed more pronounced waviness and material accumulation, highlighting the different responses of compact and foamed polymer structures to repeated thermo-mechanical ironing actions.

## 1. Introduction

Additive manufacturing (AM) has become increasingly widespread due to its design flexibility, material efficiency, and capability to produce complex geometries without tooling [1,2,3]. Researchers continuously strive to improve both the quality of 3D-printed parts and the performance of the additive manufacturing process itself. Various post-processing techniques including thermal annealing [4,5], chemical smoothing [6,7], and mechanical or laser-based surface treatments [8,9,10] have been developed to enhance surface finish, dimensional accuracy, and mechanical strength of MEX-fabricated parts. However, one of the main drawbacks of material extrusion (MEX) processes remains the presence of inter-layer micro-voids and imperfect fusion between adjacent toolpaths, which lead to reduced mechanical strength, anisotropic behavior, and compromised dimensional accuracy [11,12,13]. More detailed information about void formation mechanisms, classification, and their influence on the mechanical performance of MEX parts can be found in the comprehensive review by Tao et al. [14], which systematically analyzes the types of voids occurring in MEX structures and the parameters affecting their evolution. Over the years, several techniques have been proposed to mitigate these internal defects, such as resin infiltration [15,16], printing parameters variation [17] and the incorporation of plasticizers into the polymer matrix [18]. A promising yet scarcely explored approach for improving interlayer adhesion is the inter-layer ironing process, in which a heated nozzle applies localized pressure and thermal energy between successive layers, promoting polymer diffusion and void reduction [19,20,21,22].

To date, the scientific literature provides limited data on this process; however, sever-al studies have investigated the influence of the ironing process on the quality of additively manufactured parts produced by the MEX method, particularly regarding its effect on surface quality and dimensional accuracy.

Sardinha et al. [23,24] investigated the ironing process applied to ABS parts produced by MEX, implementing a Python-based slicing modification that enabled ironing not only on the top layer but also between selected layers. Their studies reported up to 60% reduction in surface roughness and significant mitigation of warping when ironing was applied within the first layers.

Neuhaus et al. [25] extensively investigated the ironing process applied to various thermoplastic materials, demonstrating its ability to reduce surface roughness by up to 96.6% and to improve surface compatibility for printed electronics applications.

Butt et al. [20] investigated the effect of three ironing parameters—line spacing (0.1–0.3 mm), flow rate (10–30%), and speed (50–150 mm/s)—on the dimensional accuracy, surface roughness, and Shore D hardness of MEX-printed ABS and ASA samples, concluding that ironing reduced surface roughness by up to 259% and slightly increased hardness, with ASA showing greater sensitivity to process variations than ABS.

Caputo et al. [21] conducted a fractional factorial study on MEX-printed PLA to analyze the influence of four parameters—print speed (50–100 mm/s), nozzle temperature (170–220 °C), layer thickness (0.12–0.28 mm), and infill density (5–100%)—on the surface morphology, surface roughness, and thermo-mechanical behavior of ironed samples, concluding that ironing markedly reduced surface roughness (Ra ≈ 0.8–6.5 µm).

Kechagias and Zaoutsos [26] experimentally investigated the influence of ironing parameters—flow rate (80–100%), nozzle temperature (205–230 °C), and layer thickness (0.16–0.24 mm)—on the surface texture and compression behavior of PLA components, finding that lower layer thickness and higher flow rate significantly improved the surface quality and compressive strength, with these results later confirmed by predictive additive and neural network modeling.

Alzyod et al. [27,28,29,30] conducted a series of experimental studies focused on optimizing the ironing process for MEX-printed PLA using both Box–Behnken and full factorial designs. By varying key parameters such as spacing (0.1–0.4 mm), speed (10–80 mm/s), and flow rate (5–30%), they demonstrated that ironing can substantially enhance surface quality (reducing roughness by up to 83%). Their investigations further confirmed that spacing is the most influential parameter governing surface smoothness, with optimal process settings yielding improved mechanical performance without compromising dimensional accuracy.

Pricci and Percoco [31] recently proposed a comprehensive numerical framework for analyzing the MEX ironing process in PLA parts, highlighting the influence of line spacing and ironing speed on top-layer porosity, manufacturing time, layer shape retention, and ultimate tensile strength. Their results indicated that ironing line spacing has a stronger effect on porosity reduction than ironing speed, supporting the relevance of spacing-related effects when analyzing void-related features in MEX-fabricated PLA components.

In the scientific literature, several studies have also employed the ironing process in related research areas [19,32,33,34,35], such as improving the electrical conductivity of inkjet-printed tracks on MEX substrates or enhancing the visual and surface esthetic quality of printed components, confirming the versatility of this technique beyond purely mechanical or dimensional purposes. However, despite these advances, the available literature still reveals significant gaps regarding the inter-layer ironing process, particularly its directional influence and the thermomechanical interactions occurring in other materials like lightweight or foamed polymers.

Most of the available research has focused on compact thermoplastics, whereas the application of inter-layer ironing to lightweight foamed polymers such as PLA-LW has not yet been systematically explored. Lightweight PLA (PLA-LW) is a modified grade of polylactic acid incorporating a physical blowing agent that activates at elevated extrusion temperatures, generating a microcellular structure with controlled density reduction [36,37,38]. This morphology significantly alters the rheological and thermomechanical behavior of the material, leading to reduced apparent density and lower melt viscosity compared to conventional PLA. Under optimized processing conditions, part weight can be reduced by up to 60% while maintaining adequate stiffness and dimensional stability [39]. However, the expansion and collapse of internal gas cells during deposition could introduce additional heterogeneity in interlayer bonding and surface texture, which may interact strongly with the localized pressure and heat transfer induced by ironing. Unlike conventional PLA, where ironing primarily acts on the external surface, in PLA-LW the same process could influence the internal microstructure by compacting or partially collapsing the foamed cells near the interface, altering both density and adhesion between successive layers, thus modifying the surface morphology and mechanical performance.

Despite the increasing industrial interest in lightweight extrusion-based manufacturing, a systematic investigation of inter-layer ironing applied specifically to foamed PLA materials has not yet been reported in the literature. Therefore, this study aims to experimentally evaluate the material-dependent effects of inter-layer ironing parameters, namely nozzle diameter, ironing direction, and ironing spacing, on the surface quality, Shore D hardness, surface morphology, and void-related features of MEX-printed standard PLA and pre-foamed PLA-LW components. The novelty of this work lies in the comparative experimental assessment of compact and pre-foamed PLA-based materials, with particular focus on surface morphology and microstructural changes induced by the repeated thermomechanical action of inter-layer ironing. The analysis focuses on surface roughness (Sa), Shore D hardness, surface waviness (Wa) and microscopic morphology. During preliminary observations, it was noted that the specimens exhibited visible material redistribution depending on the ironing angle. Their surface presented numerous micro-voids resembling ruptured air bubbles, likely resulting from the collapse of foamed cells during the ironing process. To systematically analyze these effects, a full factorial experimental design with 36 test specimens was employed, and for comparison, seven more test specimens without ironing or with ironing applied only on the top-most surface were produced. All tests were repeated three times for statistical reliability, resulting in 129 printed specimens for a single material, and 258 printed specimens for both materials. The experimental results were statistically analyzed through analysis of variance (ANOVA) and graphical representations of main effect trends in order to identify the most significant parameters based on *p*-values and interaction effects. Variations in nozzle diameter, ironing direction, and ironing spacing were found to influence both surface roughness (Sa) and surface waviness (Wa), suggesting that the ironing process affects surface formation through material redistribution, local density variations, and partial compaction of the foamed structure.

## 2. Experimental Setup

**Figure 1:**
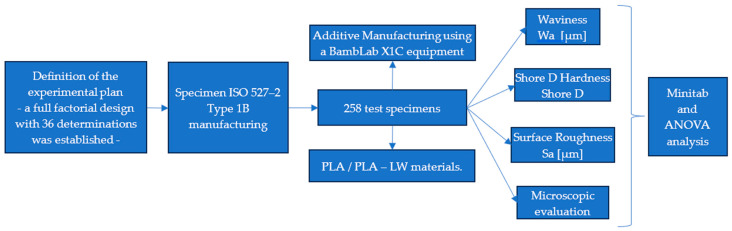
Experimental workflow.

The experimental workflow used in this study follows the sequence illustrated in Figure 1. First, the full factorial design of experiments (36 parameter combinations) was defined, including the variation in nozzle diameter, ironing direction, and ironing spacing. Based on this DOE, tensile specimens with ISO 527-2 [40,41] Type 1B geometry were manufactured on a BambuLab X1 Carbon system. In addition to the 36 configurations defined by the full factorial experimental plan, seven supplementary configurations were produced for comparison purposes, including six specimens with ironing applied only on the final surface and one specimen without ironing. Therefore, 43 configurations were obtained for each material, resulting in a total of 86 configurations for the two investigated materials.

After fabrication, each specimen was evaluated through Shore D hardness testing and surface topography analysis. The experimental results were subsequently processed using Minitab 22.5.1.0 Trial Version, generating main-effects plots, Pareto significance charts, and interaction graphs used to identify the most influential parameters and their statistical significance. All specimens were printed under controlled environmental and process conditions, maintaining constant layer height, print speed, extrusion temperature, and infill strategy in order to isolate the influence of the investigated ironing parameters. Prior to surface hardness testing, all specimens were subjected to optical microscopic examination to characterize surface morphology and detect process-induced features associated with the applied ironing conditions.

### 2.1. Experimental Plan and Specimen Manufacturing

Table 1 presents the experimental matrix corresponding to the full factorial design (4 × 3 × 3), including three factors: ironing angle—noted A (0°, 45°, 90°, 135°), nozzle diameter—noted B (0.4, 0.6, 0.8 mm), and ironing spacing—noted C (0.1, 0.2, 0.3 mm). In addition, a complementary set of specimens was manufactured without inter-layer ironing (Table 2) to provide a baseline for comparison and to evaluate the influence of the ironing strategy on surface quality.

The experimental results (Y) were further processed using the Minitab software (22.5.1.0 Trial version), which enabled the generation of main effects plots and ANOVA analysis. The statistical significance of the factors was evaluated using a significance level of *p* < 0.05, and Pareto charts were used to identify the most influential parameters affecting the investigated properties.

**Figure 2:**
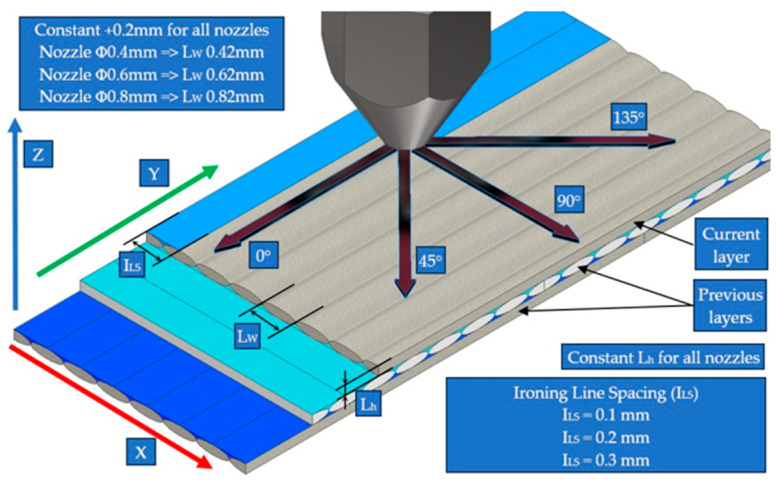
Visualization of the studied parameters.

A detailed visualization of the studied parameters and their geometric significance relative to the deposition process is illustrated in Figure 2.

The factor levels were selected to capture the influence of deposition geometry on the formation and consolidation of micro-voids during ironing. The nozzle diameters of 0.4, 0.6, and 0.8 mm were chosen because larger nozzles generate wider extrudes with greater melt volume and inherently larger micro-voids between lines, while smaller nozzles produce finer beads with smaller voids.

The ironing angles (0°, 45°, 90°, 135°) were chosen to represent the main orientations relative to the printing direction, as voids are elongated along the raster path. Applying ironing strokes parallel, perpendicular, or oblique to the deposition lines was therefore expected to produce different consolidation behaviors.

The spacing values of 0.1, 0.2, and 0.3 mm were selected to study how the overlap between successive ironing passes affects surface densification. Lower spacing provides strong overlap and consistent energy input, while higher spacing reduces coverage and may leave residual voids.

All other printing parameters were kept constant throughout the entire experimental procedure. Parameters such as layer height, extrusion temperature, build plate temperature, printing speed, infill density, infill pattern, cooling settings, and travel speed were identical for all determinations (Table 3). The line width was also maintained at a controlled offset relative to each nozzle diameter, preserving a constant +0.20 mm value (i.e., Lw = 0.42 mm for the 0.4 mm nozzle, Lw = 0.62 mm for the 0.6 mm nozzle, and Lw = 0.82 mm for the 0.8 mm nozzle).

To improve the reproducibility of the experimental procedure, the inter-layer ironing settings used during specimen fabrication are summarized in Table 4. For each ironed layer, the normal deposition toolpath was first completed, followed by a single ironing operation consisting of adjacent parallel ironing lines generated according to the selected ironing direction and spacing. No additional Z-offset was manually applied during ironing, and the nozzle-to-surface position was controlled automatically by the slicer.

**Figure 3:**
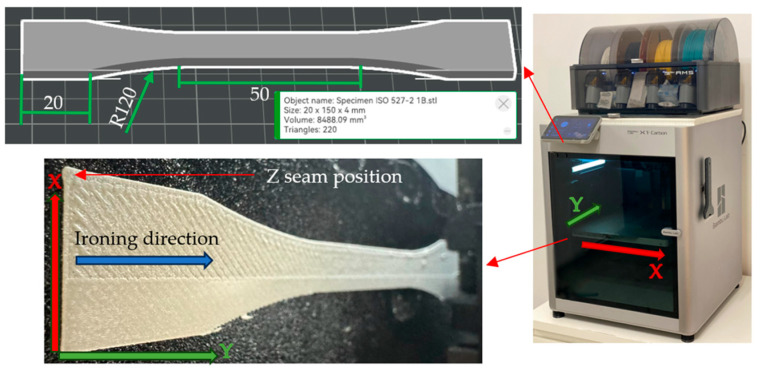
Specimen manufacturing.

The specimens, designed in accordance with the ISO 527 Type 1B tensile testing standard [40,41], were manufactured using a BambuLab X1 Carbon 3D printer (Shenzhen Tuozhu Technology Co., Ltd., Shenzhen, China) (Figure 3). Although the present study mainly focuses on surface quality, waviness, morphology, and void-related features, this standardized geometry was selected to ensure compatibility with future mechanical investigations. The geometry of the specimen was sliced following the standard dimensional requirements. Printing was performed with the specimen flat on the printing platform, aligned along the Y-axis, while the ironing direction was applied based on the selected experimental configuration.

To avoid moisture-related variability, a SUNLU FilaDryer S1 filament dryer (Zhuhai Sunlu Industrial Co., Ltd., Zhuhai, China) was used, and the filament was dried both prior to printing at 50 °C for six hours. Furthermore, during specimen fabrication, the Z-seam position was intentionally placed at the specimen corners to minimize the influence of seam-related artifacts on the evaluated surface quality and morphology. The materials used in this study were normal PLA and PLA-LW (Lightweight PLA), a foaming-modified grade of conventional PLA, both supplied by Polymaker (Changshu, China). PLA-LW was selected due to its pre-foamed microcellular structure, which results in a reduced apparent density compared with standard PLA. Although the material is already foamed, its cellular morphology may still be affected by the localized thermal and mechanical action induced during inter-layer ironing. Table 5 summarizes the main material properties as reported by the manufacturer, based on the corresponding technical and safety data sheets [42,43,44,45].

### 2.2. Measurements and Testing Equipment

**Figure 4:**
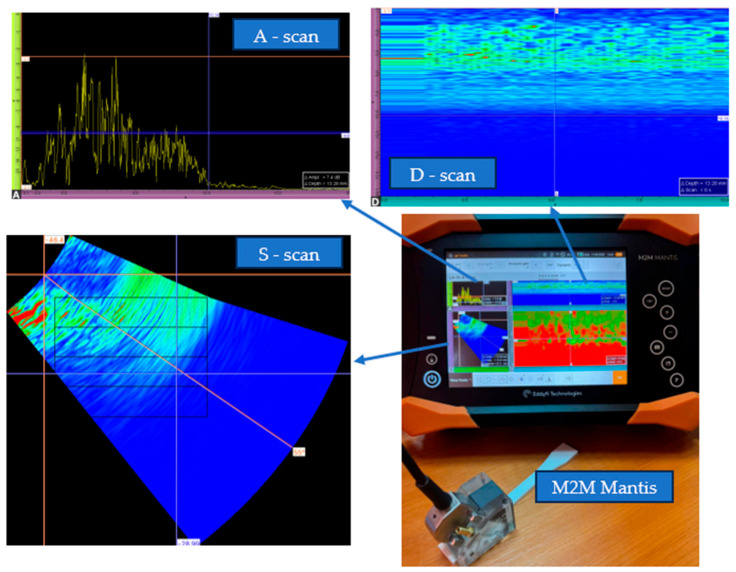
Micro-void formation evaluation.

In order to obtain preliminary information regarding the internal structure of the printed specimens, ultrasonic inspection was performed using an M2M MANTIS defectoscope (Figure 4). The inspection was carried out using a 5 MHz phased-array linear probe with 16 elements, mounted on an angled wedge marked with an incidence angle of 36.1° and an acoustic velocity of 2337 m/s; a commercial ultrasonic coupling gel was used as coupling agent. The device allows multiple scan modes (A-scan, S-scan and D-scan), enabling qualitative evaluation of wave propagation through the material. This method was used to highlight possible differences in internal density distribution and micro-void content between inter-layer ironed and non-ironed specimens. In addition, a reference specimen fabricated under identical printing conditions (Table 1, No. 1) using standard, non-foamed PLA filament was analyzed in order to provide a comparison with a material structure without foaming-induced porosity. The ultrasonic inspection was intended only for qualitative comparison, supporting the interpretation of the test results.

The testing procedure focused on surface quality, surface hardness, and morphological assessment.

**Figure 5:**
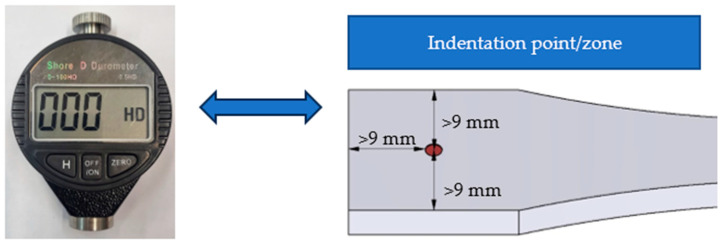
Shore D hardness evaluation.

Surface hardness was measured according to ISO 868:2003 [46], keeping the specified indentation point and specimen size (Figure 5), using a Shore D Durometer, model 560-10D, supplied by Gain Express Holdings Ltd. (Kowloon, Hong Kong), with a measuring range of 0–100 HD and a resolution of 0.5 HD.

The hardness test was included because the inter-layer ironing process may induce local material redistribution and partial collapse of the foamed microstructure specific to PLA-LW, leading to changes in apparent density and consequently in indentation hardness.

**Figure 6:**
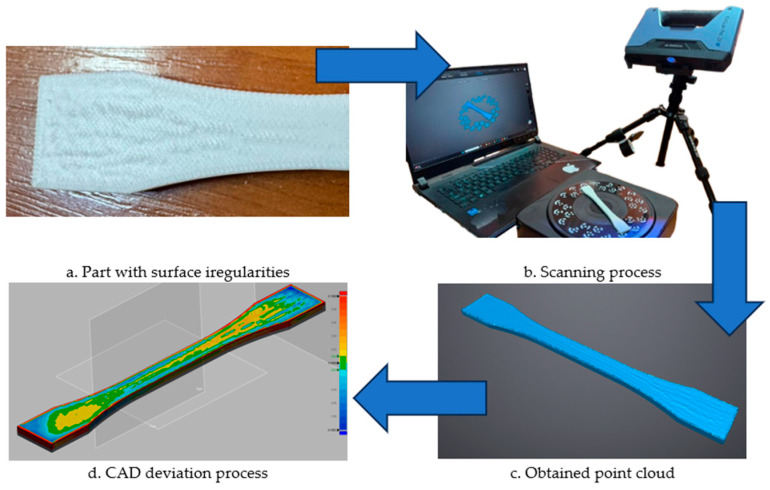
Preliminary assessment of surface deviations.

During specimen fabrication, visible surface irregularities were observed for both investigated materials. To obtain a preliminary assessment of these deviations, one representative specimen from each material was scanned (Figure 6) using an EinScan Pro 2X V3 scanner, manufactured by SHINING 3D, Hangzhou, China. The acquired point cloud was processed using EXScan Pro V4.0.1.0 software, while the deviation between the scanned geometry and the nominal CAD model was analyzed using Geomagic Design X. The comparison revealed noticeable surface deviations and waviness-like deformation patterns, particularly in the standard PLA specimens, suggesting that the ironing process may influence not only local surface roughness but also the overall surface morphology. In contrast, the PLA-LW specimen did not exhibit similarly pronounced waviness-like deformation patterns. Based on these observations, surface waviness was further investigated through topographical measurements.

**Figure 7:**
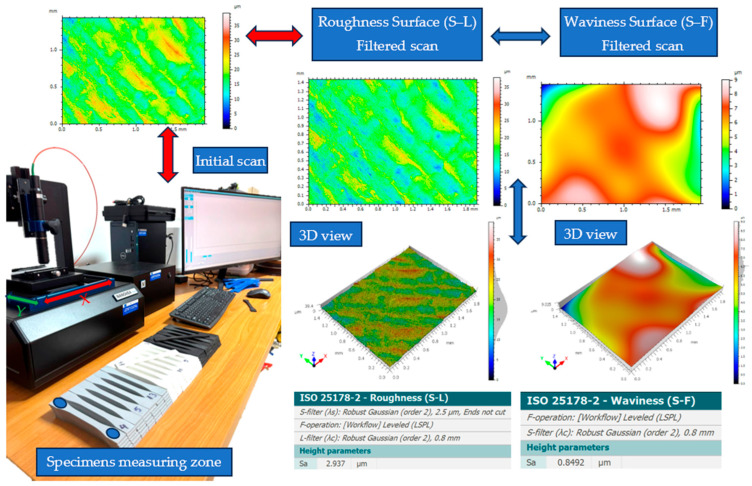
Surface roughness evaluation.

Surface topography measurements (Figure 7) were carried out using a Nanovea PS50 non-contact optical profilometer (Nanovea Inc., Irvine, CA, USA), based on chromatic confocal white-light technology for high-resolution surface characterization. The scanned area was positioned in the central region of each specimen in order to minimize edge-related effects and ensure measurement consistency. Measurements were performed over a 1.5 × 2 mm^2^ area, with a lateral step of 2 µm in both the X and Y directions, and scanning was conducted along the X-axis.

The acquired surface data were processed using Professional 3D version 10 software (Digital Surf, Besançon, France) in accordance with ISO 25178-2 [47]. For roughness evaluation, the Surface Roughness (S–L) was obtained by applying an S-filter (λs) and a Robust Gaussian L-filter (λc) to remove the short-wavelength noise, form, and waviness components. The arithmetical mean height, Sa, was selected as the representative areal roughness parameter. In addition, waviness was assessed using the Waviness Surface (S–F), obtained after applying the S-filter (λs) and removing the form component, allowing the larger-scale surface undulations to be visualized and compared among the investigated specimens.

For roughness evaluation, the Roughness Surface (S–L) was obtained by applying the appropriate filters, and the arithmetical mean height, Sa, was calculated as the representative areal roughness parameter. In addition, the Waviness Surface (S–F) was generated using the same ISO 25178-2 parameter framework, and Sa was also extracted from this filtered surface to quantify the mean height variation associated with larger-scale surface undulations.

**Figure 8:**
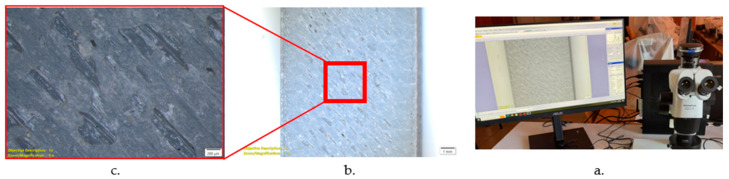
Microscopic analysis of the printed specimens: (**a**) optical microscope setup, (**b**) surface morphology (1× zoom) and (**c**) detailed view of the surface structure (5× zoom).

Microscopic analysis was performed to investigate the surface morphology of the printed specimens and to qualitatively assess the influence of the inter-layer ironing process on the visible surface features of the PLA-LW parts. Observations were carried out using an Olympus SZX10 stereo microscope (Olympus Corporation, Tokyo, Japan), equipped with a digital image acquisition system and Olympus cellSens Entry v4.3.1 software, Figure 8. Images were recorded at different magnification levels, including macro views at 1× for general inspection of the printed surface and higher magnification images at 5× for detailed observation of the surface texture, visible pores, and localized surface discontinuities. The observations focused on the distribution of surface pores, irregularities, and localized defects generated during material extrusion and inter-layer ironing. The microscopic analysis was used as a qualitative method to support the interpretation of the results obtained from surface topography measurements.

## 3. Results

**Figure 9:**
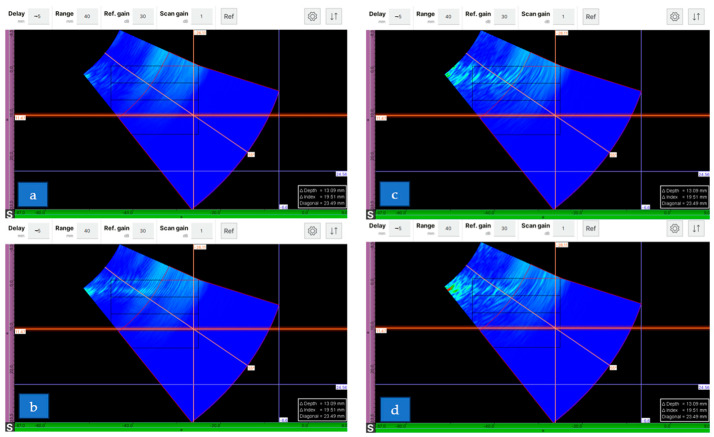
Ultrasonic S-scan comparison of the analyzed specimens: (**a**) standard PLA, angle 0°, nozzle 0.4 mm, spacing 0.1 mm; (**b**) standard PLA without ironing; (**c**) PLA-LW specimen, angle 0°, nozzle 0.4 mm, spacing 0.1 mm; (**d**) PLA-LW specimen without ironing. The color map represents relative ultrasonic signal intensity, from low-intensity regions in blue to higher-intensity regions in green/yellow/red. The S-scans were used only for qualitative comparison and not for quantitative amplitude evaluation.

The preliminary ultrasonic results for the reference and foamed specimens are shown in Figure 9, using the S-scan mode as the most representative visualization.

**Figure 10:**
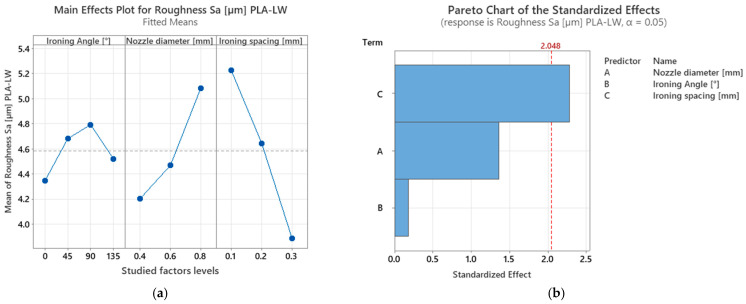
Roughness (**a**) main effects plot and (**b**) Pareto chart for PLA-LW.

As shown in Figure 10, noticeable differences can be observed between the reference PLA specimens and the PLA-LW samples. The foamed material exhibits a more heterogeneous signal distribution, which suggests the presence of internal voids generated during the foaming process, while the reference PLA specimens present a more uniform response. The comparison between ironed and non-ironed specimens indicates that the samples manufactured without inter-layer ironing tend to display a slightly more irregular signal, whereas the ironed specimens show a more uniform distribution, suggesting a partial reduction in internal discontinuities. However, the general internal structure of the PLA-LW material remains heterogeneous in both cases, indicating that the porous morphology produced by the foaming process is not completely removed by the ironing operation, which may be relevant for applications involving porous structures (e.g., scaffolds), where improved bonding between deposited layers is required without complete collapse of the internal porosity.

To further evaluate the influence of the inter-layer ironing process, the main experimental results are summarized in Table 6 and Table 7.

### 3.1. Lightweight PLA (PLA-LW)

#### 3.1.1. Roughness PLA-LW

The effect of the investigated process parameters on the surface roughness (Sa) of PLA-LW is shown in Figure 10a through the main effects plot, while the corresponding Pareto chart in Figure 10b indicates the statistically significant factors, with ironing spacing having 59.68% significance, followed by nozzle diameter with 35.68% significance and lastly, the ironing angle with 4.64% significance.

The main effects plot indicates that the ironing angle has a comparatively limited influence on surface roughness. The mean Sa value increases from approximately 4.35 μm at 0° to 4.79 μm at 90°, corresponding to an increase of approximately 10%. A moderate decrease is subsequently observed at 180°, where the mean Sa value reaches approximately 4.52 μm. However, the Pareto chart shows that the standardized effect associated with the ironing angle remains substantially below the significance threshold. Therefore, although the orientation of the ironing passes may contribute to variations in surface morphology, its influence is less pronounced than that of nozzle diameter and ironing spacing. However, the observed variations may be related to the orientation of the ironing passes relative to the deposition pattern. When the ironing direction is aligned with the deposition direction (0°), the ironing paths follow the deposited roads, facilitating the redistribution of the softened polymer and improving the filling of the gaps between adjacent filaments, which leads to a smoother surface.

**Figure 11:**
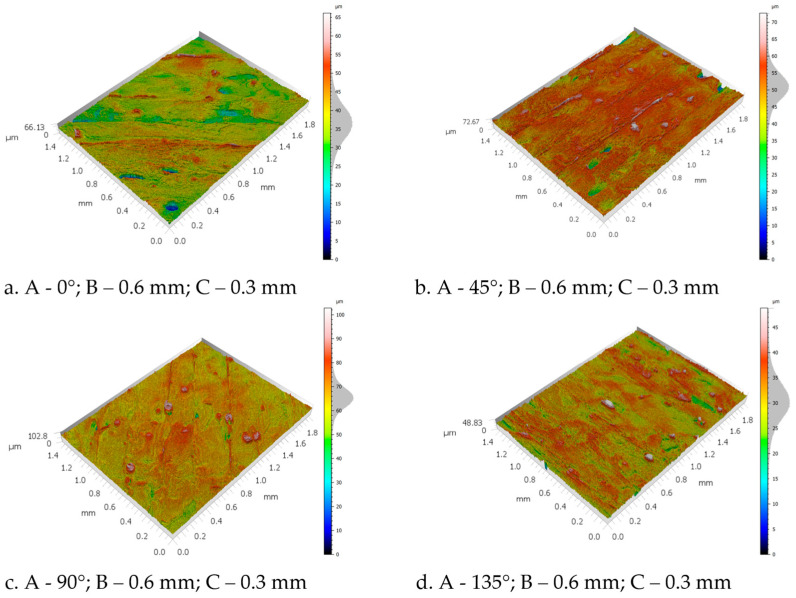
Surface roughness for PLA-LW with ironing angle variation—topographic images.

In contrast, when the ironing direction differs from the deposition orientation, the ironing passes intersect the previously deposited roads, which may generate localized scratching effects and irregular overlapping of the molten material. These observations are also confirmed by the surface images obtained from the scans. In Figure 11, the surface topographies are presented as a function of the ironing angle variation for the 0.6 mm nozzle and a spacing of 0.3 mm.

A closer examination of the surface microscopic images and topographies shows that all specimens exhibit localized surface defects, which can be associated with collapsed voids or irregularly redistributed material at the top layer. Their morphology and severity vary with the ironing angle. At 0°, the surface appears more uniform, with fewer and less pronounced depressions. At 45° and 135°, the defects become more noticeable, while the most affected surface is observed at 90°, where more pronounced irregularities and elongated surface traces can be distinguished. These visual observations are consistent with the roughness results, confirming that ironing applied perpendicular to the deposition direction leads to a less uniform surface topography.

**Figure 12:**
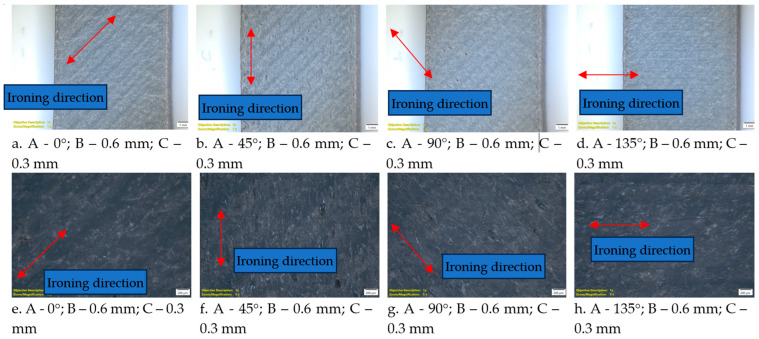
Microscopic images for PLA-LW with ironing angle variations: (**a**–**d**) 1× magnification, (**e**–**h**) 5× magnification.

Figure 12 presents the corresponding microscopic images of the topography from Figure 11 at 1× zoom also the corresponding microscopic images at 5×.

The main effects plot for the Sa parameter indicates that the nozzle diameter has a noticeable influence on the surface roughness of the printed specimens. As the nozzle diameter increases from 0.4 mm to 0.8 mm, the mean Sa value rises from approximately 4.2 μm to 5.1 μm, corresponding to an increase of about 21%. This trend is also supported by the Pareto chart, where nozzle diameter appears as a more influential factor than the ironing angle, although less significant than the ironing spacing.

**Figure 13:**
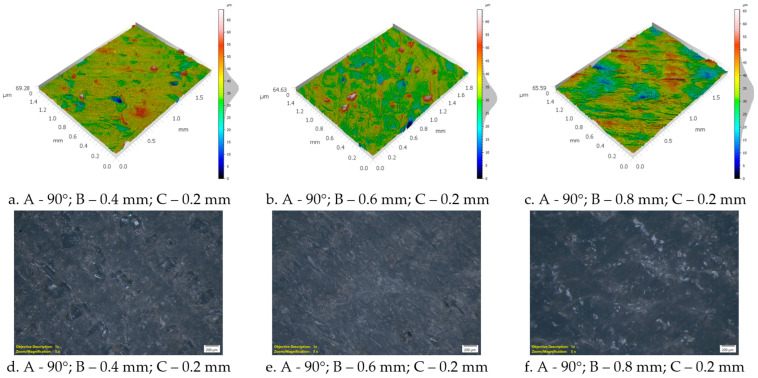
Surface roughness for PLA-LW with nozzle size variations: (**a**–**c**) topographic images, (**d**–**f**) microscopic images at 5× magnification.

The higher roughness obtained with larger nozzles may be attributed to the deposition of wider material roads and a larger volume of softened polymer at the surface. In the case of foamed materials such as PLA-LW, this effect may be amplified by the less uniform redistribution of the cellular material near the surface, leading to more pronounced height. This tendency is also supported by the surface topographies and microscopic images presented in Figure 13, which reveal a progressive deterioration of the surface morphology with increasing nozzle diameter.

For all analyzed conditions, localized defects in the form of depressions and irregular surface features can be observed, some of which may be associated with collapsed voids or non-uniform redistribution of the softened material. At 0.4 mm, the surface appears comparatively more homogeneous, with fewer but pronounced collapsed voids. As the nozzle diameter increases to 0.6 mm and 0.8 mm, the surface becomes progressively more irregular, with more visible depressions, elongated traces, and a less uniform texture.

The main effects plot for the Sa parameter shows that ironing spacing has the strongest influence on the surface roughness of PLA-LW specimens. As the spacing increases from 0.1 mm to 0.3 mm, the mean Sa value decreases markedly, from approximately 5.23 μm to 3.89 μm, corresponding to a reduction of about 26%. This trend is also confirmed by the Pareto chart, where ironing spacing is identified as the only statistically significant factor affecting surface roughness (*p*-value < 0.05).

A possible explanation is that smaller spacing values increase the overlap between adjacent ironing passes, resulting in a higher number of thermal and mechanical interactions within the same surface region. This repeated action can promote localized material accumulation, dragging of softened polymer, and the formation of surface ridges and depressions. In the case of PLA-LW, the effect may be further intensified by the cellular structure of the material. Repeated passes can locally compact or partially collapse near-surface cells, while the displaced polymer may accumulate unevenly along the ironing paths. Consequently, the surface becomes more heterogeneous, with increased height variations and higher Sa values.

By contrast, larger spacing values reduce the degree of overlap between adjacent ironing paths and limit the repeated thermo-mechanical interaction with the same surface area. This reduces excessive material redistribution and allows the near-surface cellular structure to retain a more stable morphology. As a result, the final surface becomes more uniform and the mean Sa value decreases.

**Figure 14:**
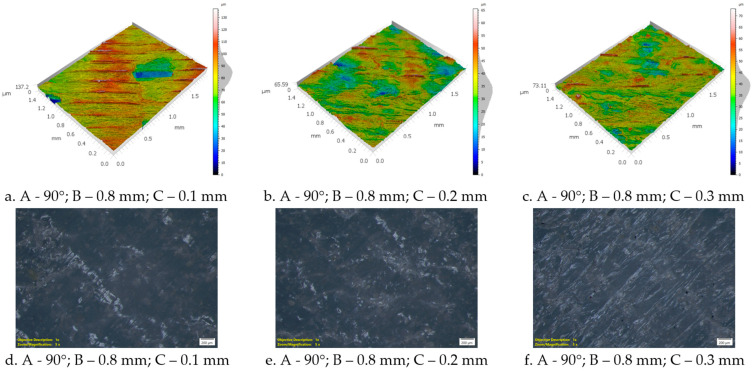
Surface roughness for PLA-LW with ironing spacing variations: (**a**–**c**) topographic images, (**d**–**f**) microscopic images at 5× magnification.

The surface topographies shown in Figure 14 further illustrate the influence of ironing spacing on surface morphology, particularly for the combination of a 90° ironing angle and a 0.8 mm nozzle diameter, where the effect is most pronounced. At the smallest spacing value of 0.1 mm, the surface exhibits the highest degree of irregularity, with visible elongated traces, localized depressions, and accumulation-related features. When the spacing increases to 0.2 mm, the severity of these defects decreases and the surface becomes more uniform. A further improvement can be observed at 0.3 mm, where the topography appears more stable and the surface irregularities are less pronounced.

**Figure 15:**
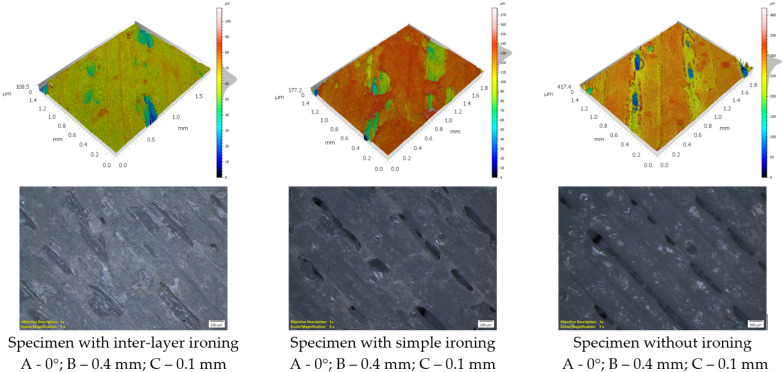
Comparison between inter-layer ironed, last layer ironed and non-ironed surfaces.

To further evaluate the effectiveness of the ironing strategy, a comparison was made between specimens manufactured using inter-layer ironing, specimens processed with ironing applied only on the final surface, and a specimen printed without ironing. The sample produced without ironing exhibited the highest Sa value, confirming the rough morphology of the as-printed top surface (Figure 15). Applying ironing only on the final layer significantly reduced the roughness; however, the specimens fabricated with inter-layer ironing generally showed lower Sa values, suggesting that repeated ironing during the build process is more effective than a single final ironing step. This behavior may be attributed to the progressive re-leveling of the deposited material throughout fabrication, which limits the accumulation of surface irregularities.

#### 3.1.2. Waviness PLA-LW

**Figure 16:**
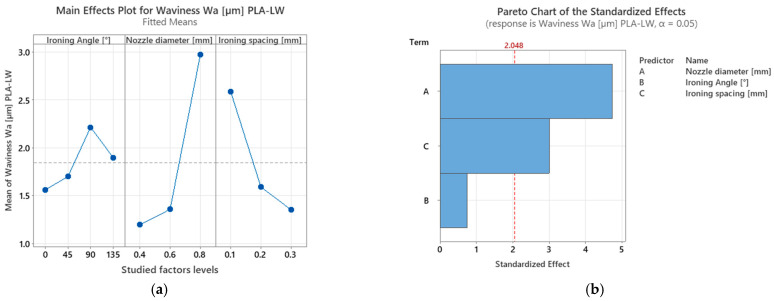
Waviness (**a**) main effects plot and (**b**) Pareto chart for PLA-LW.

Regarding surface waviness, the main effects plots presented in Figure 16 a show trends similar to those observed for surface roughness. For the ironing direction, the highest value was recorded at an angle of 90°. In the case of nozzle diameter, a substantial increase can be observed when the 0.8 mm nozzle is used compared with the other two nozzle sizes. Ironing spacing shows a decreasing trend as the spacing value increases.

However, in contrast to the roughness results, the ANOVA analysis (Figure 16b) identified that the nozzle diameter had 55.86% significance, followed by ironing spacing with 35.36% significance. For waviness, the influence of the ironing angle was not statistically significant, with only 8.78% significance.

**Figure 17:**
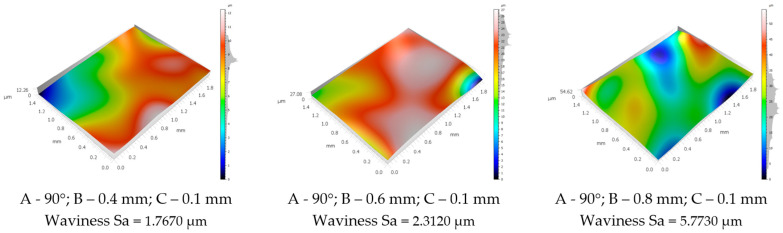
Visualization of the waviness of studied parameters for PLA-LW.

Figure 17 presents the waviness-related surface topographies obtained for different nozzle diameters at a constant ironing angle of 90° and an ironing spacing of 0.1 mm. The increase in waviness observed for the 0.8 mm nozzle may be associated with the stronger thermo-mechanical interaction between the wider nozzle and the pre-foamed PLA-LW structure. This interaction can promote non-uniform redistribution of the softened material, localized accumulation, and partial collapse of near-surface voids, all of which contribute to larger-scale height variations across the surface. Therefore, the waviness component reflects not only broad surface undulations but also the cumulative effect of collapsed voids and material accumulation generated during inter-layer ironing.

#### 3.1.3. Surface Hardness PLA-LW

**Figure 18:**
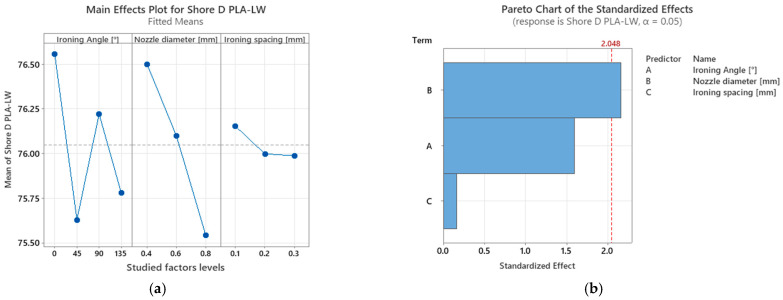
Surface (**a**) hardness main effects plot and (**b**) Pareto chart for standard PLA-LW.

The trends in the mean effects of Shore D hardness for PLA-LW are presented in Figure 18a, while the statistically significant factors are shown in Figure 18b. In contrast to the surface roughness results, the trends associated with nozzle diameter and ironing spacing are reversed. Regarding the ironing angle, the highest Shore D hardness value was recorded at an angle of 0°.

These observations may also be interpreted in relation to the cellular structure of the investigated material. Regarding nozzle diameter, this was the only factor identified as statistically significant for Shore D hardness (Figure 18b). The mean Shore D value progressively decreased by approximately 0.53% when the nozzle diameter increased from 0.4 to 0.6 mm, followed by an additional decrease of approximately 0.73% when the 0.8 mm nozzle was used. Overall, increasing the nozzle diameter from 0.4 to 0.8 mm resulted in a reduction of approximately 1.25%. Although statistically significant, the magnitude of this effect remained limited. This behavior may be associated with improved preservation of the pre-existing void structure within the pre-foamed PLA-LW filament when larger nozzle diameters are used. A smaller nozzle imposes a stronger geometrical constraint during extrusion, which may promote partial void deformation or collapse and produce a more compact deposited layer. Conversely, a larger nozzle may preserve the cellular structure to a greater extent, resulting in a less compact material volume beneath the surface and slightly lower resistance to indentation.

Ironing spacing had the smallest influence on Shore D hardness. A slight decreasing tendency was observed, with a reduction of approximately 0.20% when the spacing increased from 0.1 to 0.2 mm, followed by an additional decrease of approximately 0.02% between 0.2 and 0.3 mm.

Regarding the ironing angle, Shore D hardness did not follow a monotonic trend. The observed variations may be related to local differences in material redistribution and cellular structure deformation.

Nevertheless, a comparison between the results obtained within the experimental design and those recorded for the additional specimens indicates that inter-layer ironing increased Shore D hardness. Considering only the inter-layer-ironed specimens manufactured using the 0.4 mm nozzle, the measured increases ranged from approximately 6.85% to 10.40% relative to the non-ironed specimen, corresponding to differences of approximately 5 to 7 Shore D units. Based on the mean values, inter-layer ironing increased Shore D hardness by approximately 5.80% compared with ironing applied only on the final layer.

### 3.2. Standard PLA

#### 3.2.1. Roughness PLA

**Figure 19:**
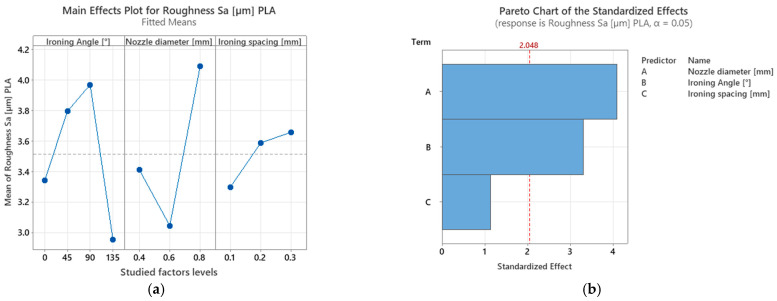
Roughness (**a**) main effects plot and (**b**) Pareto chart for standard PLA.

The main effects and Pareto charts for standard PLA are presented in Figure 19a,b. Although the magnitude of the effects differs from that observed for PLA-LW, several similar trends can be identified. However, contrary to PLA-LW, the mean Sa value increases as the ironing spacing increases. For the other studied factors, the highest roughness values are obtained for the 0.8 mm nozzle diameter and for an ironing angle of 90°.

**Figure 20:**
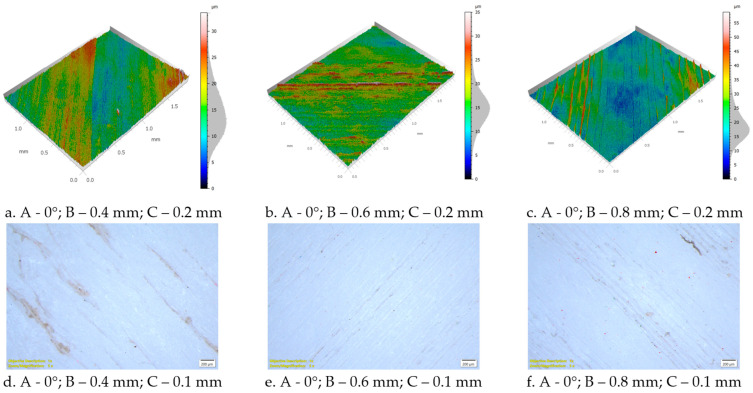
Surface roughness for standard PLA with nozzle size variations (**a**–**c**): topographic images, (**d**–**f**) microscopic images at 5× magnification.

These observations indicate that some of the process-related mechanisms affecting surface formation are common to both materials. However, based on the main effects plots and the topographical and microscopic images (Figure 20), the main difference compared with PLA-LW is related to the influence of nozzle diameter on surface roughness and ironing spacing.

For standard PLA, nozzle diameter was identified as the most statistically significant factor (*p* < 0.05), with 47.93% significance, followed by ironing angle with 38.74% significance and lastly, the ironing spacing with 13.33% significance.

For the nozzle diameter, the main effects plot reveals a non-linear variation in surface roughness. The lowest mean Sa value was obtained using the 0.6 mm nozzle, whereas the 0.4 mm nozzle resulted in a moderately higher roughness value, corresponding to an increase of approximately 10.70% relative to the 0.6 mm nozzle. The 0.8 mm nozzle produced the highest surface roughness, with an increase of approximately 34.29% compared with the 0.6 mm nozzle.

This behavior suggests that the intermediate nozzle diameter provides a more favorable balance between surface leveling and material redistribution. By contrast, the smaller nozzle may promote localized material accumulation and waviness-related irregularities, whereas the larger nozzle generates wider deposited roads and more pronounced surface deviations.

**Figure 21:**
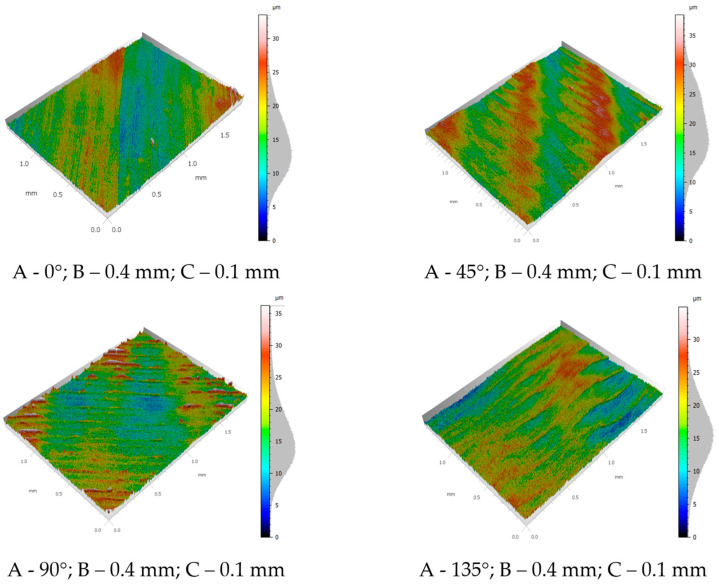
Surface topographies for standard PLA with ironing angle variations.

**Figure 22:**
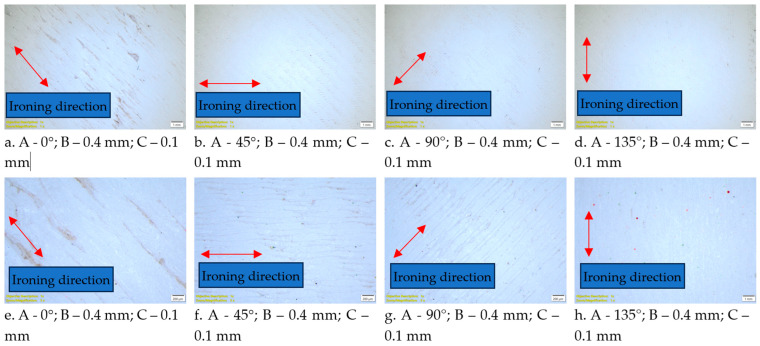
Microscopic images for standard PLA with ironing angle variations: (**a**–**d**) 1× magnification, (**e**–**h**) 5× magnification.

The influence of the ironing angle was also above the statistical significance threshold, suggesting a relevant effect on surface roughness. Figure 21 presents the surface topographies obtained through surface scanning, while Figure 22 presents the microscopic images at 1× and 5× magnification for ironing angle variations. The images reveal direction-dependent surface features whose orientation is generally consistent with the ironing path.

The main effects plot indicates a non-linear variation in surface roughness as a function of the ironing angle. Increasing the ironing angle from 0° to 45° resulted in an increase in Sa of approximately 13.60%, followed by a further increase of 4.58% between 45° and 90°. The highest mean Sa value was obtained at an ironing angle of 90°. Increasing the ironing angle to 135° led to a decrease of approximately 25.54% relative to the value obtained at 90°. Nevertheless, the Sa value recorded at 135° remained approximately 1.54% lower than that obtained at 0°.

More pronounced and widely distributed traces can be observed when the ironing passes intersect the deposited roads transversely, particularly at 90°, indicating less uniform redistribution and localized overlapping of the softened material. By contrast, the surfaces obtained at 0° and 135° appear comparatively more uniform, although isolated defects and directional traces remain visible.

#### 3.2.2. Waviness Standard PLA

**Figure 23:**
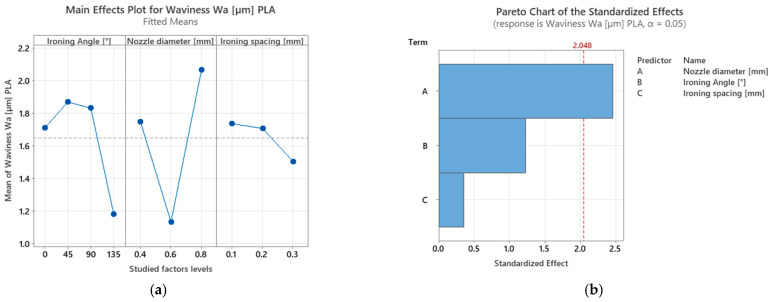
Waviness (**a**) main effects plot and (**b**) Pareto chart for PLA.

For standard PLA, the statistically significant factor was only the nozzle diameter, with the greatest influence on waviness, accounting for 60.88% of the total effect, followed by ironing angle, with a contribution of 30.42%. The influence of the ironing spacing was considerably lower, accounting for only 8.71%, and was not statistically significant. Regarding the variation in the mean effects, in contrast to PLA-LW, standard PLA exhibited substantially higher surface waviness when the 0.4 mm nozzle was used. This behavior was also observed during the microscopic analysis. The main effects plots presented in Figure 23 also show different tendencies when the ironing angle was varied. More specifically, waviness increased by 9.24% between 0° and 45°, followed by a small decrease of 2.015% between 45° and 90°, and a further decrease of 35.47% between 90° and 135°.

**Figure 24:**
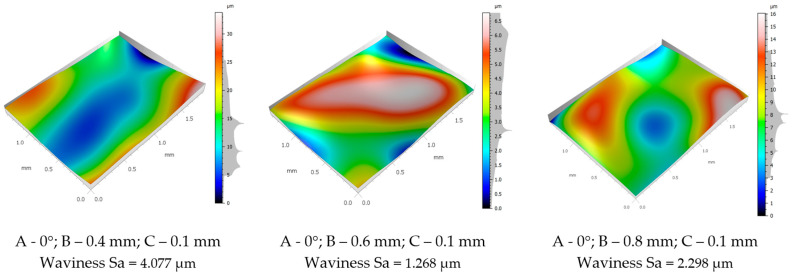
Visualization of the waviness of studied parameters for standard PLA.

Both the main effects plots and the topographic (Figure 24) and microscopic images (Figure 20) suggest that the highest waviness values were obtained when the 0.8 mm nozzle was used. A decrease of 35.06% was recorded when the nozzle diameter was increased to 0.6 mm, followed by a substantial increase of approximately 82.02% when the 0.8 mm nozzle was used. While the trends are different from that observed for PLA-LW, both materials presented the highest waviness at 0.8 mm nozzle diameter.

Unlike pre-foamed PLA-LW, standard PLA does not exhibit a pre-existing cellular structure. Therefore, the surface morphology is not governed by the preservation, deformation, or local collapse of internal cells to the same extent. Instead, the repeated thermomechanical action of the nozzle may lead more directly to local material redistribution and accumulation. For standard PLA, the higher waviness observed when using the 0.4 mm nozzle may be associated with the cumulative redistribution of material induced by inter-layer ironing. At a constant ironing spacing, the smaller nozzle requires a larger number of adjacent passes, increasing the degree of overlap between ironing tracks. Each pass may generate minor local material accumulations, which can be transferred to the subsequent layer and progressively amplified throughout the printing process. This layer-by-layer propagation of surface irregularities may explain the higher waviness Sa values recorded for the 0.4 mm nozzle.

Increasing the nozzle diameter to 0.6 mm reduces the number of ironing passes and limits excessive overlapping, resulting in a more uniform surface. However, when the 0.8 mm nozzle is used, the wider ironing tracks may redistribute a larger amount of material during each pass, leading to a slight increase in waviness compared with the 0.6 mm nozzle. Nevertheless, the Waviness Sa values remain lower than those obtained with the 0.8 mm nozzle.

A similar mechanism may explain the effect of ironing spacing. Larger spacing values reduce the number of overlapping ironing passes and limit the progressive accumulation of material between consecutive layers, resulting in lower surface waviness.

#### 3.2.3. Surface Hardness for Standard PLA

**Figure 25:**
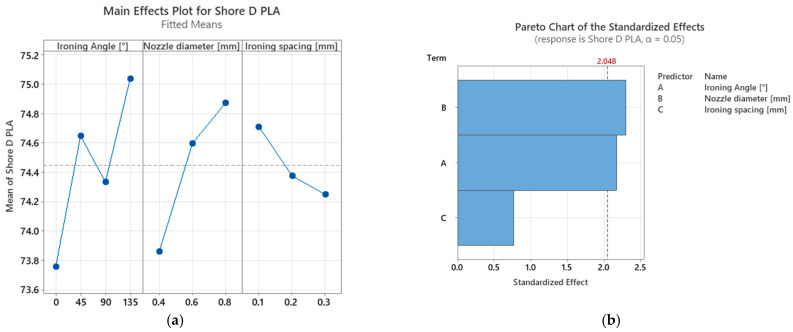
Surface (**a**) hardness main effects plot and (**b**) Pareto chart for standard PLA.

The Pareto chart indicates that nozzle diameter had the strongest influence on the Shore D hardness of standard PLA specimens, with 43.89% influence, followed by the ironing angle with 41.48%, Figure 25. Both factors exceeded the statistical significance threshold, whereas the effect of ironing spacing remained below the critical value with 14.63% influence.

In contrast to pre-foamed PLA-LW, standard PLA does not contain a pre-existing cellular structure. Therefore, its behavior is not primarily governed by the preservation, deformation, or local collapse of internal voids within the filament. The overall variations in surface hardness within the experimental plan remained relatively small, with a maximum of approximately 1.73%. Increasing the nozzle diameter from 0.4 to 0.6 mm increased the Shore D hardness by approximately 1.00%, followed by a further increase of 0.37% when the 0.8 mm nozzle was used. Overall, hardness increased by approximately 1.37% between the 0.4 and 0.8 mm nozzles. This limited increase may be associated with differences in near-surface consolidation, as wider deposited paths may provide more continuous support beneath the area subjected to indentation.

The ironing angle also had a statistically significant but non-linear influence, with the lowest mean value obtained at 0° and the highest at 135°. By contrast, ironing spacing produced a slight decrease of approximately 0.61% between 0.1 and 0.3 mm and was not statistically significant.

The topographic parameters exhibited a different, non-linear response to nozzle diameter. Surface waviness decreased by approximately 35.06% between 0.4 and 0.6 mm and then increased by 82.02% when the 0.8 mm nozzle was used. Similarly, surface roughness decreased by approximately 10.70% between 0.4 and 0.6 mm and increased by 34.29% between 0.6 and 0.8 mm. Within the investigated range, the 0.6 mm nozzle therefore provided the most favorable balance between surface smoothing and material redistribution. The use of the 0.8 mm nozzle may have promoted local material accumulation and the propagation of surface undulations between consecutive layers.

### 3.3. Final Discussions

To summarize the results, Table 8 presents the direction and magnitude of the variations in the mean effects values for each investigated parameter. The percentages reported for the intermediate levels were calculated relative to the preceding factor level.

The results indicate that the investigated ironing parameters had a considerably stronger influence on surface morphology than on Shore D hardness. Ironing spacing produced pronounced but material-dependent effects. For PLA-LW, increasing the spacing from 0.1 to 0.3 mm reduced surface roughness by 25.61% and surface waviness by 47.71%. For standard PLA, the same increase led to a 10.87% rise in roughness and a 13.39% reduction in waviness. Increasing the distance between adjacent passes may therefore limit large-scale surface irregularities, although the reduced overlap can also decrease the local smoothing effect in standard PLA.

The effect of nozzle diameter also depended strongly on the material type. For pre-foamed PLA-LW, increasing the nozzle diameter from 0.4 to 0.8 mm increased roughness by 20.98% and waviness by 148.50%. Standard PLA exhibited a non-linear response: when the nozzle diameter increased from 0.4 to 0.6 mm, roughness decreased by 10.70% and waviness by 35.06%. Both parameters then increased when the 0.8 mm nozzle was used, by 34.29% and 82.02%, respectively. These differences may be associated with the pre-existing cellular structure of PLA-LW, which can be preserved, deformed, or partially collapsed during deposition and ironing. The ironing angle also generated non-linear variations. Between the first and last investigated levels, PLA-LW exhibited increases of 4.02% in roughness and 21.44% in waviness, whereas standard PLA showed reductions of 11.54% and 30.93%, respectively. Compared with the topographic parameters, Shore D hardness exhibited only limited variations, with overall changes below approximately 2%. Thus, the studied parameters primarily affected the surface morphology rather than hardness.

To provide a descriptive comparison between the investigated ironing strategies, Table 9 presents the results obtained for the non-ironed specimens, the specimens subjected to ironing only on the final layer, and the inter-layer-ironed specimens. Since all additional specimens were manufactured using the 0.4 mm nozzle, only the corresponding results from the factorial experimental plan were considered for the inter-layer ironing strategy. The mean values for inter-layer ironing were calculated from the 12 combinations obtained with the 0.4 mm nozzle, whereas the mean values for final-layer ironing were calculated from the six additional specimens. The percentage variations were determined relative to the non-ironed specimen and, additionally, between the two ironing strategies.

For standard PLA, the non-ironed specimen exhibited a roughness value of 9.070 µm. By comparison, the mean Sa value obtained for the inter-layer-ironed specimens manufactured with the 0.4 mm nozzle was approximately 3.410 µm, corresponding to a reduction of approximately 62.41%. A similar tendency was observed for PLA-LW, for which Sa decreased from 21.84 µm for the non-ironed specimen to an average value of approximately 4.203 µm for the inter-layer-ironed specimens, representing a reduction of approximately 80.76%. These results indicate that inter-layer ironing can substantially reduce small-scale surface irregularities, particularly in the case of pre-foamed PLA-LW.

Regarding the influence of inter-layer ironing on surface waviness, the mean Waviness Sa value for PLA-LW decreased from 3.544 µm for the non-ironed specimen to approximately 1.197 µm for the inter-layer-ironed specimens produced with the 0.4 mm nozzle, corresponding to a reduction of approximately 66.22%. For standard PLA, the mean waviness value also decreased from 2.977 to approximately 1.749 µm, representing a reduction of approximately 41.26%. However, certain parameter combinations produced higher waviness values than the non-ironed specimen. For example, when a 0.4 mm nozzle, a 90° ironing angle, and a 0.2 mm ironing spacing were used, the Waviness Sa value increased from 2.977 to 3.868 µm. This behavior suggests that, for standard PLA, the repeated thermomechanical action associated with inter-layer ironing may promote local material accumulation and the layer-by-layer propagation of surface undulations under specific processing conditions.

The Shore D hardness results further highlight the differences between the investigated ironing strategies. For standard PLA, the mean Shore D value increased from 71.17 for the non-ironed specimen to 72.67 for the specimens subjected to ironing only on the final layer and to 73.86 for the inter-layer-ironed specimens. These values correspond to increases of approximately 2.10% and 3.78%, respectively, relative to the non-ironed specimen. The effect was more pronounced for pre-foamed PLA-LW, for which the Shore D hardness increased from 70.50 for the non-ironed specimen to an average value of 72.31 when ironing was applied only on the final layer, corresponding to an increase of approximately 2.56%. By contrast, the mean Shore D value obtained for the inter-layer-ironed specimens reached 76.50, representing an increase of approximately 8.51% relative to the non-ironed specimen and 5.80% compared with final-layer ironing. This behavior may be associated with the progressive compaction of the pre-existing cellular structure and the partial deformation or collapse of internal voids induced by the repeated thermomechanical action of the nozzle after each deposited layer.

The reliability of the results is supported by their agreement with trends reported in the literature, at least for standard PLA, since comparable data for pre-foamed PLA-LW remain limited. In the present study, the non-ironed PLA specimen exhibited Sa = 9.070 µm, while inter-layer ironing resulted in values ranging from 2.372 to 5.382 µm. Although previous studies generally report the profile parameter Ra rather than the areal parameter Sa, the values are comparable in magnitude. Alzyod et al. reported a reduction in Ra from 8.829 to 2.730 µm after optimization of inter-layer ironing parameters [28].

A similar agreement was observed for ironing spacing. Increasing the spacing from 0.1 to 0.3 mm increased the mean Sa value for standard PLA by 10.87%. Alzyod et al. also associated greater spacing with higher roughness values, as the reduced overlap between adjacent paths limits uniform surface smoothing [28]. By contrast, Shore D hardness varied only slightly: increasing the spacing from 0.1 to 0.3 mm reduced hardness by approximately 0.61%. Similarly, Butt et al. reported limited variations in hardness associated with ironing parameters, although their study considered other polymers [20].

## 4. Conclusions

The present work systematically evaluated the effects of inter-layer ironing on components fabricated from standard PLA and lightweight PLA (PLA-LW). The experimental results provide valuable insights into the role of this processing approach, allowing the following key conclusions to be established.

Inter-layer ironing substantially influenced the surface morphology of both standard PLA and pre-foamed PLA-LW specimens. Compared with the non-ironed parts, ironing after each deposited layer reduced surface roughness by up to 85.22% and increased Shore D hardness by up to 10.40%. These maximum variations were observed for individual PLA-LW specimens manufactured using the 0.4 mm nozzle. However, the effect on surface waviness depended strongly on the material and the selected process parameters.

Ironing spacing produced a clear but material-dependent effect. For PLA-LW, increasing the spacing from 0.1 to 0.3 mm reduced surface roughness by 25.61% and surface waviness by 47.71%. For standard PLA, the same change increased roughness by 10.87%, while waviness decreased by 13.39%. Increasing the distance between adjacent passes may therefore limit large-scale surface irregularities, although the reduced overlap can also decrease the local smoothing effect.

The influence of nozzle diameter also depended on the material type. For PLA-LW, increasing the diameter from 0.4 to 0.8 mm increased roughness by 20.98% and waviness by 148.50%. This behavior may be associated with the preservation, deformation, or partial collapse of the pre-existing cellular structure during extrusion and ironing.

Standard PLA exhibited a non-linear response. The lowest mean roughness and waviness values were obtained using the 0.6 mm nozzle. Compared with the 0.4 mm nozzle, it reduced roughness by 10.70% and waviness by 35.06%. When the diameter increased from 0.6 to 0.8 mm, both parameters increased again, by 34.29% and 82.02%, respectively. Within the investigated range, the 0.6 mm nozzle therefore provided the most favorable balance between surface smoothing and material redistribution.

The ironing angle generated non-linear variations for both materials, indicating that the relative orientation of the ironing paths can influence local material redistribution and the resulting surface morphology.

Shore D hardness exhibited smaller variations within the factorial experimental plan, with overall changes below approximately 2%. Nevertheless, inter-layer ironing increased hardness relative to the non-ironed parts, particularly for PLA-LW. Based on the mean values, Shore D hardness increased by 8.51% compared with the non-ironed specimen and by 5.80% compared with final-layer ironing. This effect may be related to the progressive compaction and partial deformation of the cellular structure caused by the repeated thermomechanical action of the nozzle.

The results demonstrate that the optimal inter-layer ironing parameters cannot be transferred directly from standard PLA to pre-foamed PLA-LW. The response of standard PLA is mainly governed by material redistribution, whereas PLA-LW is additionally influenced by its pre-existing cellular morphology.

Although the present study provides a detailed experimental assessment of the influence of inter-layer ironing parameters on the surface quality and Shore D hardness of PLA and PLA-LW parts, some limitations should be acknowledged. The investigation was limited to two PLA-based materials, one specimen geometry, and a fixed set of printing parameters, while only the ironing angle, nozzle diameter, and ironing spacing were varied. In addition, the mechanical evaluation was focused on Shore D hardness, without including tensile, flexural, or impact testing.

Future work should extend the analysis to other thermoplastic and foamed materials, different specimen geometries, and additional process parameters, such as ironing temperature, ironing speed, flow rate, and layer height. Further investigations should also include mechanical testing, thermal analysis, and more detailed microstructural characterization in order to better correlate ironing-induced surface modifications with the overall performance of MEX-printed parts.

## Figures and Tables

**Table 1:** Experimental matrix for the 36-specimen factorial plan.

No.	A—Ironing Angle [°]	B—Nozzle Diameter [mm]	C—Ironing Spacing [mm]	Y (Results)
1	0	0.4	0.1	Y1
2	0	0.4	0.2	Y2
3	0	0.4	0.3	Y3
4	0	0.6	0.1	Y4
5	0	0.6	0.2	Y5
6	0	0.6	0.3	Y6
7	0	0.8	0.1	Y7
8	0	0.8	0.2	Y8
9	0	0.8	0.3	Y9
10	45	0.4	0.1	Y10
11	45	0.4	0.2	Y11
12	45	0.4	0.3	Y12
13	45	0.6	0.1	Y13
14	45	0.6	0.2	Y14
15	45	0.6	0.3	Y15
16	45	0.8	0.1	Y16
17	45	0.8	0.2	Y17
18	45	0.8	0.3	Y18
19	90	0.4	0.1	Y19
20	90	0.4	0.2	Y20
21	90	0.4	0.3	Y21
22	90	0.6	0.1	Y22
23	90	0.6	0.2	Y23
24	90	0.6	0.3	Y24
25	90	0.8	0.1	Y25
26	90	0.8	0.2	Y26
27	90	0.8	0.3	Y27
28	135	0.4	0.1	Y28
29	135	0.4	0.2	Y29
30	135	0.4	0.3	Y30
31	135	0.6	0.1	Y31
32	135	0.6	0.2	Y32
33	135	0.6	0.3	Y33
34	135	0.8	0.1	Y34
35	135	0.8	0.2	Y35
36	135	0.8	0.3	Y36

**Table 2:** Specimens without inter-layer ironing.

Specimen No.	Ironing Angle [°]	Nozzle Diameter [mm]	Ironing Spacing [mm]
1.1	0	0.4	0.1
1.2	0	0.4	0.2
1.3	0	0.4	0.3
1.4	45	0.4	0.1
1.5	90	0.4	0.1
1.6	135	0.4	0.1
1.7	-	0.4	-

**Table 3:** Constant parameters.

Parameter	Value	Parameter	Value
Printing temperature	220 °C	Build platform temperature	50 °C
Infill	100%	Layer height	0.3 mm
Infill pattern	Rectilinear	Wall number	2
Printing speed	300 mm/s	Retraction	6.5 mm

**Table 4:** **Ironing process** constant parameters.

Parameter	Value
Ironing temperature	220 °C
Number of ironing passes	1 pass per layer/per selected surface
Ironing flow rate	10%
Ironing pattern	rectilinear
Ironing speed	300 mm/s
Ironing inset	0 mm

**Table 5:** Material properties.

Material	Density (g/cm^3^)	Glass Transition Temperature (°C)	Melt Index (g/10 min)	Melting Temperature (°C)	Crystallization Temperature (°C)
PLA-LW	0.90	58.7	9.21	150.8	114.0
PLA	1.17	61.0	7–11	150.0	122.2

**Table 6:** Experimental results for the 36-specimen factorial plan.

No.	A	B	C	Sa Roughness PLA	Wa Waviness PLA	SD PLA	SD PLA-LW	Sa Roughness PLA-LW	Wa Waviness PLA-LW
1	0	0.4	0.1	2.564	2.227	73.50	76.17	4.899	1.022
2	0	0.4	0.2	2.550	0.719	72.17	77.33	5.931	1.365
3	0	0.4	0.3	3.996	1.031	71.83	77.00	4.114	1.076
4	0	0.6	0.1	2.692	1.268	74.50	77.17	3.666	1.364
5	0	0.6	0.2	3.313	1.351	74.50	76.17	3.682	0.921
6	0	0.6	0.3	3.854	1.625	73.67	75.17	3.863	1.765
7	0	0.8	0.1	3.670	2.298	73.67	77.00	5.949	3.349
8	0	0.8	0.2	3.813	2.105	75.50	76.83	3.343	1.251
9	0	0.8	0.3	3.610	2.799	74.50	76.17	3.673	1.945
10	45	0.4	0.1	3.390	2.007	75.00	75.33	3.227	0.654
11	45	0.4	0.2	4.150	3.081	73.83	76.33	3.885	0.899
12	45	0.4	0.3	3.633	2.010	72.67	75.50	3.275	0.725
13	45	0.6	0.1	2.924	0.782	75.17	76.00	5.810	1.391
14	45	0.6	0.2	3.008	0.740	76.50	75.83	7.365	1.156
15	45	0.6	0.3	3.114	0.797	74.00	75.83	5.277	0.804
16	45	0.8	0.1	4.996	3.372	74.33	76.17	5.502	6.238
17	45	0.8	0.2	4.835	2.292	75.50	74.67	4.126	1.867
18	45	0.8	0.3	4.100	1.757	74.83	75.00	3.669	1.566
19	90	0.4	0.1	3.149	1.995	75.17	77.83	5.121	1.767
20	90	0.4	0.2	5.382	3.868	72.83	76.83	3.733	1.487
21	90	0.4	0.3	4.203	1.380	74.00	77.67	3.981	1.374
22	90	0.6	0.1	2.811	0.576	74.67	76.00	5.372	2.312
23	90	0.6	0.2	2.666	0.577	73.17	76.33	4.035	1.076
24	90	0.6	0.3	3.385	1.815	75.00	75.00	3.680	1.497
25	90	0.8	0.1	4.673	1.629	75.17	74.33	7.724	5.773
26	90	0.8	0.2	4.825	2.897	73.50	76.33	4.818	3.007
27	90	0.8	0.3	4.621	1.764	75.50	75.67	4.677	1.644
28	135	0.4	0.1	2.372	0.573	74.67	75.67	4.664	1.596
29	135	0.4	0.2	2.517	0.827	75.17	75.67	4.231	0.972
30	135	0.4	0.3	3.010	1.266	75.50	76.67	3.376	1.430
31	135	0.6	0.1	2.959	2.312	74.83	77.67	4.646	2.455
32	135	0.6	0.2	2.933	0.802	74.17	74.83	3.032	0.962
33	135	0.6	0.3	2.879	0.978	75.00	77.17	3.208	0.596
34	135	0.8	0.1	3.385	1.815	75.83	74.50	6.120	3.120
35	135	0.8	0.2	3.055	1.238	75.67	74.83	7.567	4.131
36	135	0.8	0.3	3.483	0.838	74.50	75.00	3.850	1.810

**Table 7:** Results for the specimens without inter-layer ironing.

No.	A	B	C	Sa Roughness PLA	Sa Waviness PLA	SD PLA	SD PLA-LW	Sa Roughness PLA-LW	Sa Waviness PLA-LW
1.1	0	0.4	0.1	4.324	1.353	72.83	72.83	11.99	1.695
1.2	0	0.4	0.2	5.655	0.754	72.67	72.50	7.295	1.025
1.3	0	0.4	0.3	8.639	1.768	71.50	73.00	5.548	1.421
1.4	45	0.4	0.1	2.262	0.954	72.50	72.00	11.94	2.164
1.5	90	0.4	0.1	4.073	1.257	73.50	72.17	14.53	2.731
1.6	135	0.4	0.1	4.957	0.884	73.00	71.33	12.70	2.880
1.7	-	0.4	-	9.070	2.977	71.17	70.50	21.84	3.544

**Table 8:** Results variation for every level of the studied factors.

Parameter		Ironing Angle [°]		Nozzle Diameter [mm]		Ironing Spacing [mm]	
		PLA—LW	PLA	PLA—LW	PLA	PLA—LW	PLA
Level		Variation	Variation	Variation	Variation	Variation	Variation
Roughness Sa [µm]	1	-	-	-	-	-	-
	2	↑ 7.710%	↑ 13.60%	↑ 6.340%	↓ 10.70%	↓ 11.09%	↑ 8.745%
	3	↑ 2.380%	↑ 4.584%	↑ 13.76%	↑ 34.29%	↓ 16.33%	↑ 1.954%
	4	↓ 5.670%	↓ 25.54%	-	-	-	-
Total		↑ 4.020%	↓ 11.54%	↑ 20.98%	↑ 19.92%	↓ 25.61%	↑ 10.87%
Waviness Sa [µm]	1	-	-	-	-	-	-
	2	↑ 8.830%	↑ 9.236%	↑ 13.46%	↓ 35.06%	↓ 38.49%	↓ 1.710%
	3	↑ 30.31%	↓ 2.015%	↑ 119.0%	↑ 82.02%	↓ 14.99%	↓ 11.88%
	4	↓ 14.37%	↓ 35.47%	-	-	-	-
Total		↑ 21.44%	↓ 30.93%	↑ 148.5%	↑ 18.20%	↓ 47.71%	↓ 13.39%
Shore D	1	-	-	-	-	-	-
	2	↓ 1.210%	↑ 1.210%	↓ 0.530%	↑ 1.000%	↓ 0.200%	↓ 0.450%
	3	↑ 0.780%	↓ 0.420%	↓ 0.730%	↑ 0.370%	↓ 0.020%	↓ 0.170%
	4	↓ 0.580%	↑ 0.950%	-	-	-	-
Total		↓ 1.020%	↑ 1.730%	↓ 1.250%	↑ 1.370%	↓ 0.220%	↓ 0.610%

**Table 9:** Comparison between non-ironed, final-layer-ironed, and inter-layer-ironed specimens manufactured using the 0.4 mm nozzle.

Material	Parameter	No Ironing	Final-Layer Ironing, Mean	Inter-Layer Ironing, Mean	Final-Layer vs. No Ironing	Inter-Layer vs. No Ironing	Inter-Layer vs. Final-Layer
PLA	Roughness Sa [µm]	9.070	4.599	3.410	↓ 45.04%	↓ 62.41%	↓ 31.60%
PLA	Waviness Sa [µm]	2.977	1.162	1.749	↓ 60.98%	↓ 41.26%	↑ 50.53%
PLA	Shore D	71.17	72.67	73.86	↑ 2.100%	↑ 3.78%	↑ 1.640%
PLA-LW	Roughness Sa [µm]	21.84	10.667	4.203	↓ 51.16%	↓ 80.76%	↓ 60.60%
PLA-LW	Waviness Sa [µm]	3.544	1.986	1.197	↓ 43.96%	↓ 66.22%	↓ 39.72%
PLA-LW	Shore D	70.50	72.31	76.50	↑ 2.560%	↑ 8.510%	↑ 5.800%

## Data Availability

The original contributions presented in this study are included in the article. Further inquiries can be directed to the corresponding authors.
